# Aggressiveness of care in the last days of life in the emergency department of a tertiary hospital in Korea

**DOI:** 10.1186/s12904-022-00988-3

**Published:** 2022-06-07

**Authors:** Jung Sun Kim, Sun Young Lee, Min Sung Lee, Shin Hye Yoo, Jeongmi Shin, Wonho Choi, Yejin Kim, Hyung Sook Han, Jinui Hong, Bhumsuk Keam, Dae Seog Heo

**Affiliations:** 1 Department of Internal Medicine, Seoul National University Hospital, Seoul, Korea; 2 Public Healthcare Center, Seoul National University Hospital, Seoul, Korea; 3 Department of Emergency Medicine, Seoul National University Hospital, Seoul, Korea; 4 Center for Palliative Care and Clinical Ethics, Seoul National University Hospital, 101 Daehak-ro, Jongno-gu, Seoul, 03080 Korea; 5 Patient-Centered Clinical Research Coordinating Center, National Evidence-Based Healthcare Collaborating Agency, Seoul, Korea

**Keywords:** End-of-life care, Emergency department, Disease-related deaths

## Abstract

**Background:**

High-quality end-of-life (EOL) care requires both comfort care and the maintenance of dignity. However, delivering EOL in the emergency department (ED) is often challenging. Therefore, we aimed to investigate characteristics of EOL care for dying patients in the ED.

**Methods:**

We conducted a retrospective cohort study of patients who died of disease in the ED at a tertiary hospital in Korea between January 2018 and December 2020. We examined medical care within the last 24 h of life and advance care planning (ACP) status.

**Results:**

Of all 222 disease-related mortalities, 140 (63.1%) were men, while 141 (63.5%) had cancer. The median age was 74 years. As for critical care, 61 (27.5%) patients received cardiopulmonary resuscitation, while 80 (36.0%) received mechanical ventilation. The absence of serious illness (*p* = 0.011) and the lack of an advance statement (*p* < 0.001) were both independently associated with the receipt of more critical care. Only 70 (31.5%) patients received comfort care through opioids. Younger patients (< 75 years) (*p* = 0.002) and those who completed life-sustaining treatment legal forms (*p* = 0.001) received more comfort care. While EOL discussions were initiated in 150 (67.6%) cases, the palliative care team was involved only in 29 (13.1%).

**Conclusions:**

Patients in the ED underwent more aggressive care and less comfort care in a state of imminent death. To ensure better EOL care, physicians should minimize redundant evaluations and promptly introduce ACP.

**Supplementary Information:**

The online version contains supplementary material available at 10.1186/s12904-022-00988-3.

## Background

A “good death” consists of several essential conditions, including the protection of dignity, free of pain and suffering, and the ability to be among loved ones [1, 2]. When death is imminent, it is thus imperative to decide upon the scope of medical care. Here, physicians should work toward the goals expressed by dying persons [3], including their preferred places of death [4–7].

The emergency department (ED) is not typically the desired place of death [7, 8]. While ED physicians prioritize life by pursuing resuscitation and stabilization, some patients die on site. ED mortality was around 0.3% in Western countries, mainly due to cardiovascular problems [9–11]. In Korea, the national systematic database showed 0.7% of disease-related mortality in the ED from 2017 to 2019 [12].

Dying in the ED should be avoided due to the difficulty of ensuring dignity and comfort; there is insufficient privacy and time for providing end-of-life (EOL) communication [13, 14]. Even with the high demand for comfort care, dying patients are often not rated as a priority to healthcare providers in ED [15–17]. Furthermore, the coronavirus disease of 2019 (COVID-19) pandemic has also imposed strict quarantine policies such as large-scale screening for viral symptoms and visitor restrictions [18], thus making the ED more unsuitable for death [19, 20]. Although the total number of ED visitations decreased during the pandemic in the United States [21, 22] and Korea [23, 24], the ED mortality rate significantly increased after COVID-19 in Korea [25]. Hence, there is growing interest in providing and even initiating palliative care for terminal patients in ED [26–30].

Meanwhile, ‘Act on Decisions on Life-Sustaining Treatment (LST) for Patients in Hospice and Palliative Care or at the End of Life (hereafter, LST Decision Act)’ came into force in Korea in 2018 to protect the dignity and value of human beings in the EOL [31] which had been easily overlooked. Accordingly, it highlighted the need to address advance care planning (ACP) and issues related to LST in the EOL care context. However, there is a lack of evidence on the state of medical care provided under constraints such as EDs, especially in Korea; nor are there major discussions on EOL care strategies in the ED [32–35].

We conducted this study to clarify the characteristics of EOL care for actively dying patients in the ED at a tertiary hospital in Korea and the relevant factors in receiving critical care and comfort care.

## Methods

### Study design and setting

This is a single-center, retrospective cohort study of adult patients who died of disease in the ED at Seoul National University Hospital (SNUH) between January 2018 and December 2020. SNUH is a 1,761-bed tertiary referral hospital in Korea that employs 1,947 total doctors, most of whom work in acute and specialized care. Of note, there were no inpatient hospice-palliative care units in the hospital. There are approximately 70,000 visits to the ED each year at SNUH, with 65 doctors and 37 beds available in the adult unit. Of all deaths during the assessment period, we first selected those that occurred among individuals ≥ 19 years of age in the ED; we then excluded patients who were already dead or on cardiopulmonary resuscitation (CPR) upon arrival (i.e., out-of-hospital cardiac arrest) and who died from non-disease-related causes (i.e., unknown or trauma).

### Data collection

We reviewed electronic medical records to obtain data on demographics (i.e., age, sex, type of health insurance), comorbidities (i.e., cancer, other serious illnesses) via the Charlson Comorbidity Index [36], and variables related to the ED visit (i.e., chief complaints, prior place before ED visit, acuity at triage according to the Korean Triage and Acuity Scale (KTAS) [37], ED visit time, time of death). We then examined the critical care/detailed medical procedures provided in the ED. We collected data on ACP status, including documents from the electrical medical records and database associated with the National Agency for Management of Life-Sustaining Treatment of the Korea National Institute for Bioethics Policy. We conducted all procedures according to the principles of the Declaration of Helsinki. The Institutional Review Board of SNUH reviewed and approved the study protocol (no. H-2104–102-1212). Informed consent requirements were waived.

### Definitions and measurements

The chief complaint is a primary reason for the ED visit, with categories of neurological, cardiopulmonary, gastrointestinal, genitourinary, constitutional, or others. To evaluate patients’ acuity levels, KTAS was used, which was developed in 2012. It is a symptom-oriented tool that investigates the patients’ symptoms, with primary (characteristics common to most symptoms and signs such as consciousness, blood pressure, heart rate, respiration rate, fever, pain, presence of hemorrhage, and trauma) and secondary factors (characteristics applied to specific symptoms). Then, the well-trained ED staff uses to assess the critical first look of the patients at triage. It is a five-level triage scale ranging from KTAS 1, which requires immediate aggressive treatment due to life-threatening conditions, to KTAS 5, a non-emergency visit due to chronic illness. Considering the study population, we further grouped them as those at risk of death without immediate conditions (i.e., levels 1 and 2) and others (i.e., levels 3 to 5) [37]. Additionally, the length of stay refers to the time interval between the initial ED visit and death.

According to the LST Decision Act, a patient with adequate decision-making capacity can choose not to receive LST at the EOL via an advance statement, in either advance directives or Physicians Orders for LST. On the other hand, if the patient cannot express an intention at the time of the decision, family members’ surrogate decision-making should be done by prioritizing the best interests of the patient and by considering previously known values and preferences of the patient. We regarded the former as self-determination and the latter as family determination, and the legal forms (hereafter, LST legal forms) were necessary for both. Without the complete legal forms, withholding or withdrawing LST from patients are not protected by the law, which may be burdensome for physicians in the decision-making.

We defined *critical care* as receiving any of the following in the last 24 h of life: CPR, mechanical ventilation (MV), renal replacement therapy (RRT), and extracorporeal membrane oxygenation (ECMO). Moreover, we regarded patients within the following criteria of serious medical conditions [38] as having a serious illness: cancer with distant metastases, a chronic obstructive pulmonary disease with oxygen demand or in need of hospitalization, end-stage renal disease on dialysis, congestive heart failure in need of hospitalization, liver cirrhosis in Child–Pugh class C, diabetes with severe complications (ischemic heart disease, peripheral vascular disease, and renal disease), amyotrophic lateral sclerosis, or dementia with evidence of illness or advanced disease.

### Statistical analysis

We used descriptive data to summarize the demographic and clinical characteristics. We applied the Pearson’s chi-squared test or Fisher’s exact test for the categorical variables and used the analysis of variance for the mean length of stay in the ED to compare groups by year. We also calculated the proportions for each type of critical care, procedure, diagnostic evaluation, and medication. We conducted a stepwise forward-selection multivariable logistic regression analysis to identify relevant factors in critical care/comfort care, considering our observations in the univariable analyses. All statistical analyses were two-sided (statistical significance at *p*-values < 0.05, 95% confidence interval; CI). We conducted all analyses using STATA version 16.0 (StataCorp LP, College Station, TX).

## Results

### Patient characteristics

Of the 3,549 deaths at SNUH between January 2018 and December 2020, 222 died of disease-related causes in the ED, other than trauma or unknown causes, and they were eligible for the final analysis (Supplementary Fig. 1). There were 481 all-cause deaths or 0.33% (481/145,901) of all ED visitations, and 55.4% of deaths occurred in 2020 (Table 1).

**Table 1:** Baseline characteristics for patients who died in the emergency department by year

Variables	Total n (%)	2018 n (%)	2019 n (%)	2020 n (%)	*p*-value
**Disease-related deaths**/Total deaths	46.2% (222/481)	44.6% (41/92)	43.6% (58/133)	48.0% (123/256)	0.667
**Total deaths**/No. of patients who visited the ED	0.33% (481/145,901)	0.17% (92/52,789)	0.26% (133/52,064)	0.62% (256/41,048)	< 0.001
**Age (years), median (range)**	74 (36–100)	75 (47–94)	73 (38–100)	74 (36–94)	0.164
< 75	118 (53.15)	21 (51.22)	31 (53.45)	66 (53.66)	0.908
≥ 75	104 (46.85)	20 (48.78)	27 (46.55)	57 (46.34)	
**Sex**					
Male	140 (63.06)	27 (65.85)	37 (63.79)	76 (61.79)	0.889
Female	82 (36.94)	14 (34.15)	21 (36.21)	47 (38.21)	
**Health insurance**					
National Health Insurance	198 (89.19)	35 (85.37)	56 (96.55)	107 (86.99)	0.105
Medicaid/None	24 (10.81)	6 (14.63)	2 (3.45)	16 (13.01)	
**Serious illness**^***a***^					
Yes	182 (81.98)	39 (95.12)	46 (79.31)	97 (78.86)	0.053
No	40 (18.02)	2 (4.88)	12 (20.69)	26 (21.14)	
**Cancer**					
Yes	141 (63.51)	23 (56.10)	39 (67.24)	79 (64.23)	0.510
No	81 (36.49)	18 (43.90)	19 (32.76)	44 (35.77)	
**Chief complaint**^***b***^					
Neurological	76 (34.23)	11 (26.83)	21 (36.21)	44 (35.77)	0.199
Cardiopulmonary	74 (33.33)	17 (41.46)	13 (22.41)	44 (35.77)	
Gastrointestinal	36 (16.22)	6 (14.63)	15 (25.86)	15 (12.20)	
Constitutional/genitourinary/others	36 (16.22)	7 (17.07)	9 (15.52)	20 (16.26)	
**Place prior to ED visit**					
Home	153 (68.92)	29 (70.73)	37 (63.79)	87 (70.73)	0.618
Others	69 (31.08)	12 (29.27)	21 (36.21)	36 (29.27)	
**KTAS level**					
1–2	194 (87.39)	35 (85.37)	45 (77.59)	114 (92.68)	0.015
3–5	28 (12.61)	6 (14.63)	13 (22.41)	9 (7.32)	
**Length of stay (minutes),** median (Q1-Q3)	733 (326–1479)	306 (119–810)	605 (336–1158)	981 (426–1892)	< 0.001

*Abbreviations*: *ECOG* Eastern Cooperative Oncology Group, *ED* Emergency department, *KTAS* Korean Triage and Acuity Scale, *No*. Number

^*a*^ Patients were considered to have serious illness if they were diagnosed as any of the followings [38]: cancer with distant metastases, a chronic obstructive pulmonary disease with oxygen demand or in need of hospitalization, end-stage renal disease on dialysis, congestive heart failure in need of hospitalization, liver cirrhosis in Child–Pugh class C, diabetes with severe complications (ischemic heart disease, peripheral vascular disease, and renal disease), amyotrophic lateral sclerosis, or dementia with evidence of illness or advanced disease

^*b*^ Only one priority cause of visits was selected as the chief complaint. Neurological symptoms: altered mentality; Cardiopulmonary: dyspnea, cough, sputum, chest pain; Gastrointestinal: abdominal pain, hematemesis, melena, hematochezia, nausea, vomiting, diarrhea; genitourinary: dysuria, hematuria, frequency; Constitutional: fever, chills, general weakness, poor oral intake, hypotension; Others: in-hospital cardiac arrest (*n* = 1), cancer pain (*n* = 1), local wound discharge (*n* = 1), levin-tube insertion (*n* = 1)

The median patient age was 74 years (range, 36–100), with 140 (63.1%) men. Upon arrival, 82.0% of patients had serious illnesses, with 63.5% (141/222) having advanced cancer. The chief complaints were neurological (34.2%), cardiopulmonary (33.3%), and gastrointestinal (16.2%) in order. Neurological and cardiopulmonary symptoms consistently accounted for more than 50% (over 70% in 2020). Further, 68.9% arrived at the ED directly from home, with 87.4% deemed KTAS level of 1–2 at triage. The median length of stay was 733 min (12.2 h), increasing from 306 min (5.1 h) in 2018 to 981 min (16.4 h) in 2020 (*p* < 0.001) (Table 1).

### Medical care in the last 24 h of ED visits

**Fig. 1:**
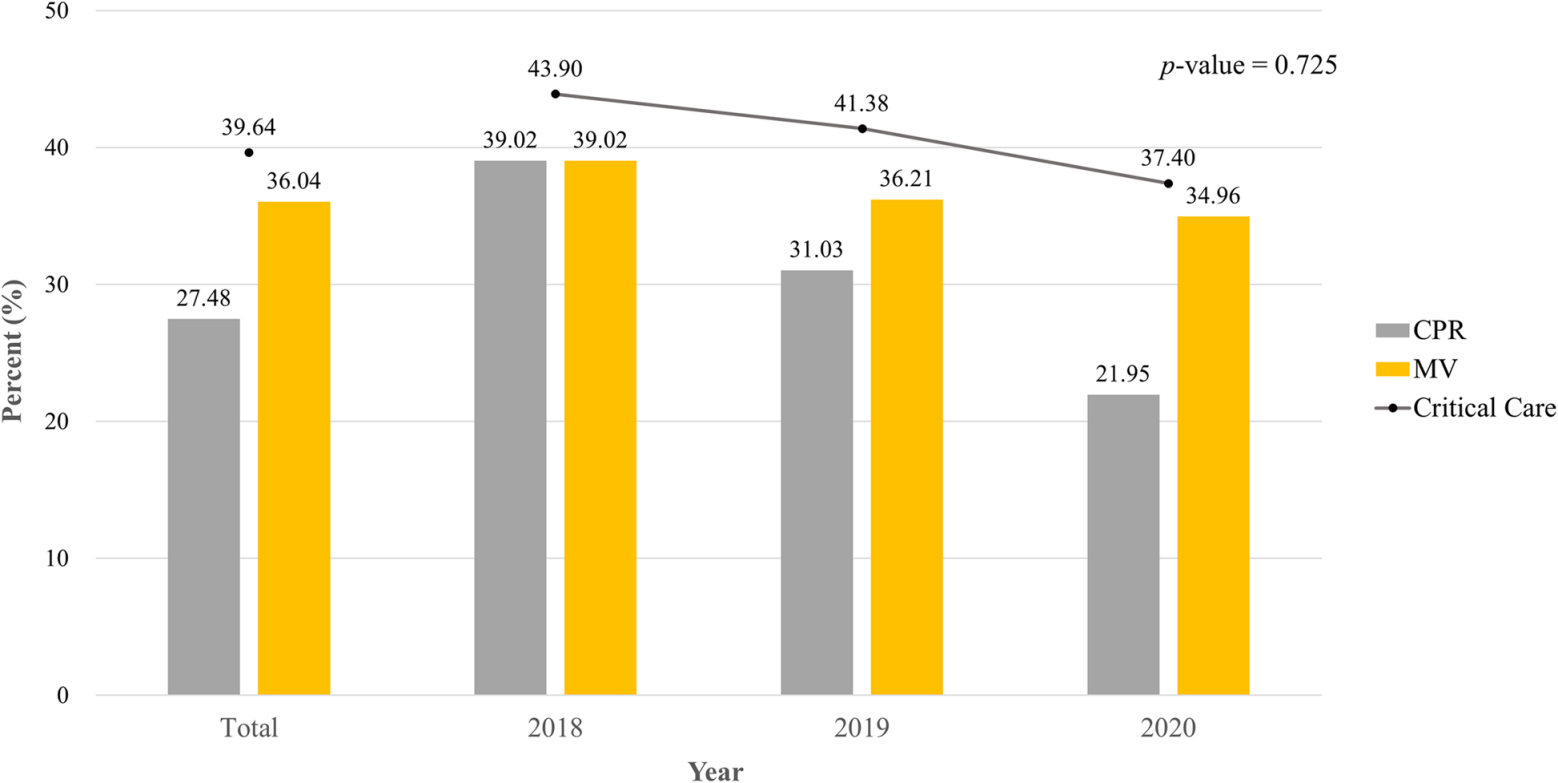
Status of critical care in the emergency department in the last 24 h of life by year. Abbreviations: CPR, cardiopulmonary resuscitation; MV, mechanical ventilation

**Fig. 2:**
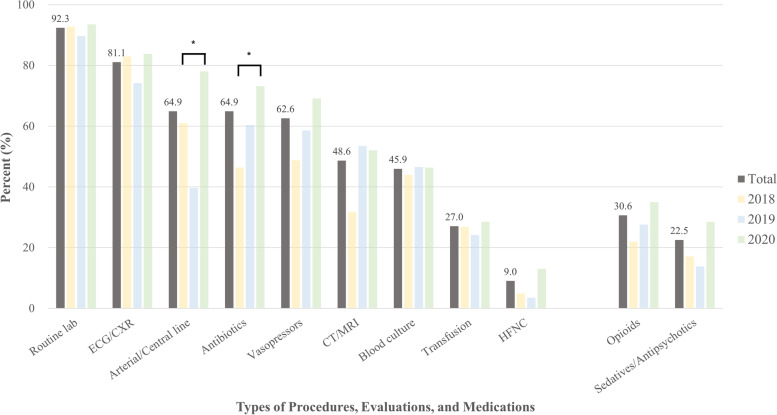
Status of general medical care (procedures, evaluations, and medications) in the emergency department in the last 24 h of life by year. Abbreviations: CXR, chest radiograph; CT, computed tomography; ECG, electrocardiogram; HFNC, high-flow nasal cannula; MRI, magnetic resonance imaging. ^*^p-value < 0.05

We found that 40% (88/221) of those with disease-related deaths received critical care in their last 24 h; the overall proportion steadily decreased from 43.9% in 2018 to 37.4% in 2020, despite no significant difference. Specifically, 61 (27.5%) and 80 (36.0%) patients underwent CPR and MV, respectively. Only one patient each received RRT (0.5%) and ECMO (0.5%) (Fig. 1, Supplementary Table 1). The majority had drawn blood for laboratory testing (92.3%) and underwent electrocardiogram/chest radiograph (81.1%), while most received antibiotics (64.9%) or vasopressors (62.6%). By contrast, less than one-third (31.5%) received opioids, while less than one-quarter (22.5%) received sedatives/antipsychotics on their last day (Fig. 2, Supplementary Table 1).

Compared to the 120 with LST legal forms, significantly higher proportions of the 102 who had not completed and registered the forms beforehand underwent CPR (47.1% vs. 10.8%, *p* < 0.001) and MV (46.1% vs. 27.5%, *p* = 0.004). While patients with the forms received more antibiotics (71.7% vs. 56.8%, *p* = 0.021) and opioids (40.8% *vs.* 20.6%, *p* = 0.001), there were no intergroup differences in other medical care/evaluations (Supplementary Fig. 2).

### ACP status

The ACP status (e.g., initiation of conversation, advance statement, legal form on LST, and palliative care consultation) is demonstrated in Supplementary Table 2. The percentage of patients with ACP conversations before death significantly increased over time, reaching 93.5% in 2020. While still under 30%, the proportion who initiated discussions before their final ED visit also increased. Moreover, advance statements increased from 9.8% in 2018 to 33.3% in 2020. Among the 60 with advance statements, 41.7% made them after ED visitation.

**Fig. 3:**
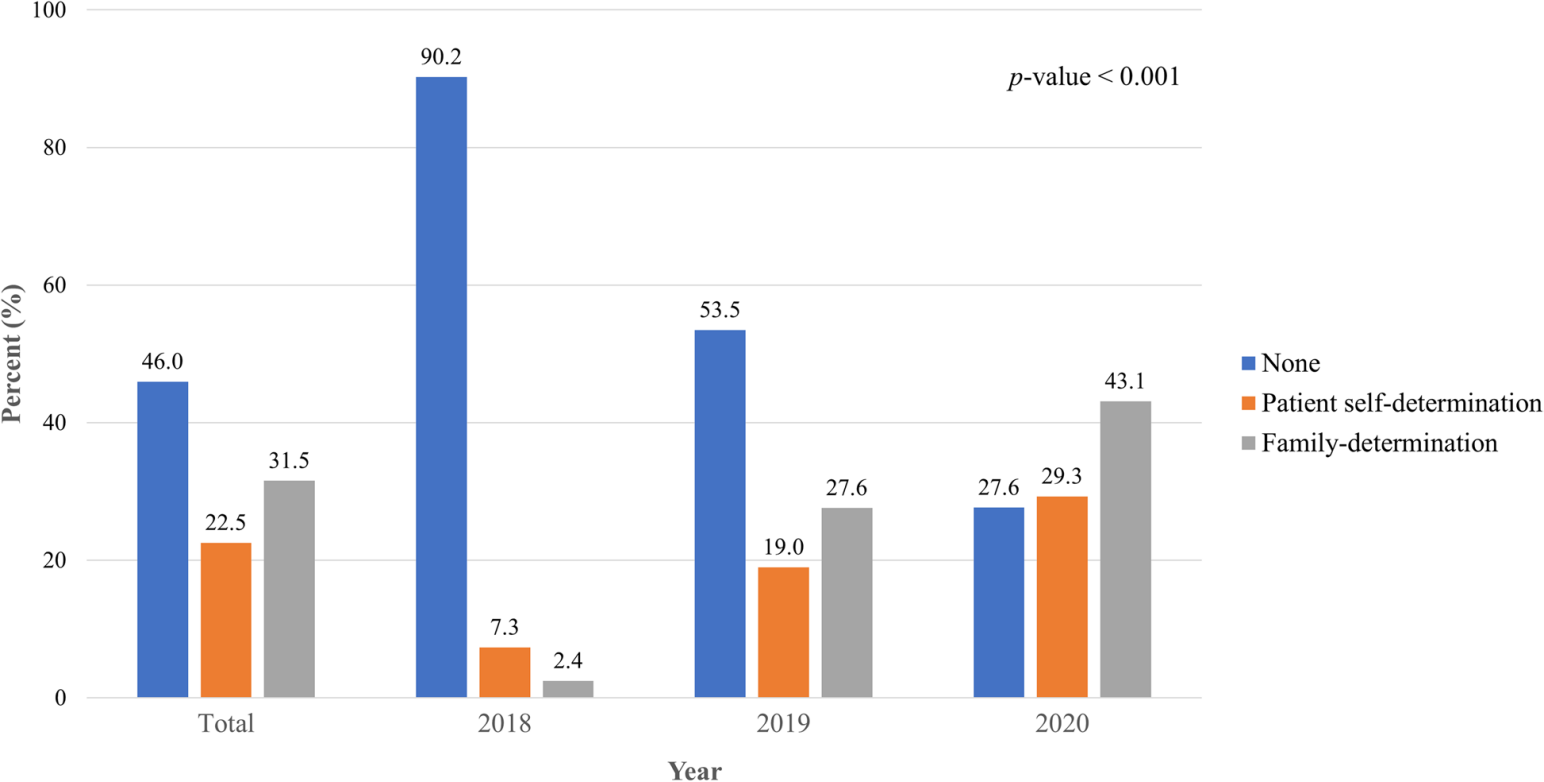
Trends in the proportions (%) of decision-makers in legal form documentations on life-sustaining treatment by year

The number of patients who died without LST legal forms significantly decreased from 2018 to 2020 (90.2% to 27.6%). While the self-determination rate increased from 7.3% to 29.3%, the family determination rate increased from 2.4% to 43.1% (Fig. 3). When analyzed by time, 85% (102/120) of those with LST forms made submissions after ED visitation. There were more self-determinations than family determinations (72.2% *vs.* 27.8%) when LST forms were completed before ED visitation, and vice versa if after (36.3% *vs.* 63.7%).

Overall, 13.1% of patients received palliative care consultations; while this proportion steadily increased from 9.8% in 2018 to 14.6% in 2020, the difference was not significant (*p* = 0.700) (Supplementary Table 2).

### Factors associated with critical care

Patients without advanced cancer (51.9% *vs.* 32.6%, *p* = 0.005; odds ratio [OR] 2.22, 95% CI 1.27–3.89) or serious illness (62.5% *vs.* 34.6%, *p* = 0.001; OR 3.14, 95% CI 1.55–6.40) received significantly more critical care than those with.

ACP conversation status was also associated with critical care, as 17.0% and 36.7% of patients with conversations before and after ED visits received critical care, respectively (OR 2.82, 95% CI 1.23–6.47). All patients without conversations received critical care.

Patients without advance statements (49.4% *vs.* 13.3%, *p* < 0.001; OR 6.34, 95% CI 2.83–14.19) or LST legal forms (52.0% *vs.* 29.2%, *p* = 0.001; OR 2.63, 95% CI 1.51–4.57) received significantly more critical care than those with. While patients without palliative care consultations were more likely to receive critical care (43.0% *vs.* 17.2%, *p* = 0.005; OR 3.62, 95% CI 1.33–9.89), this was not influenced by age, sex, health insurance type, place before ED visitation, or KTAS (Table 2).

**Table 2:** Factors associated with receiving critical care at end-of-life in the emergency department

	**No Critical Care** **(*N* = 134)**	**Critical Care** **(*N* = 88)**		**Univariate** **Logistic Regression**
**Variables**	**n (%)**	**n (%)**	***p*-value**^***b***^	**OR (95% CI)**
**Age (years)**				
≥ 75	61 (58.65)	43 (41.35)	0.626	ref
< 75	73 (61.86)	45 (38.14)		0.87 (0.51–1.50)
**Sex**				
Female	50 (60.98)	32 (39.02)	0.886	ref
Male	84 (60.00)	56 (40.00)		1.04 (0.60–1.82)
**Health insurance**				
Medicaid/None	17 (70.83)	7 (29.17)	0.267	ref
National Health Insurance	117 (59.09)	81 (40.91)		1.68 (0.67–4.24)
**Serious illness**^*a*^				
Yes	119 (65.38)	63 (34.62)	0.001	ref
No	15 (37.50)	25 (62.50)		3.14 (1.55–6.40)
**Cancer (active)**				
Yes	95 (67.38)	46 (32.62)	0.005	ref
No	39 (48.15)	42 (51.85)		2.22 (1.27–3.89)
**Place prior to ED visit**				
Others	43 (62.32)	26 (37.68)	0.689	ref
Home	91 (59.48)	62 (40.52)		1.13 (0.63–2.02)
**KTAS level**				
3–5	15 (53.57)	13 (46.43)	0.432	ref
1–2	119 (61.34)	75 (38.66)		0.73 (0.33–1.61)
**Advance care planning conversation**				
Before ED visit	39 (82.98)	8 (17.02)	< 0.001	ref
None	0 (0.00)	25 (100.00)		1
After ED visit	95 (63.33)	55 (36.67)		2.82 (1.23–6.47)
**Advance statement**				
Yes	52 (86.67)	8 (13.33)	< 0.001	ref
No	82 (50.62)	80 (49.38)		6.34 (2.83–14.19)
**Legal form documentation for LST implementation**				
Yes	85 (70.83)	35 (29.17)	0.001	ref
No	49 (48.04)	53 (51.96)		2.63 (1.51–4.57)
**Palliative care consultation**				
Yes	24 (82.76)	5 (17.24)	0.005	ref
No	110 (56.99)	83 (43.01)		3.62 (1.33–9.89)

*Abbreviations*: *ECOG* Eastern Cooperative Oncology Group, *ED* Emergency department, *EF* Ejection fraction, *FEV1* Forced expiratory volume in one second, *KTAS* Korean Triage and Acuity Scale, *LST* Life-sustaining treatment, *NYHA* New York Heart Association

^*a*^ Patients were considered to have serious illness if they were diagnosed as any of the followings [38]: cancer with distant metastases, a chronic obstructive pulmonary disease with oxygen demand or in need of hospitalization, end-stage renal disease on dialysis, congestive heart failure in need of hospitalization, liver cirrhosis in Child–Pugh class C, diabetes with severe complications (ischemic heart disease, peripheral vascular disease, and renal disease), amyotrophic lateral sclerosis, or dementia with evidence of illness or advanced disease

^*b*^* p*-values were calculated using Pearson’s chi-squared test for age, sex, health insurance, serious illness, cancer, prior place to ED visit, advance statement, legal form documentation and palliative consultation, or Fisher’s exact test for ACP conversation

According to the multivariable logistic regression analysis, patients without serious illness (adjusted OR 2.62, 95% CI 1.25–5.50) and/or without advance statements (adjusted OR 5.77, 95% CI 2.56–13.03) received more critical care in their last 24 h (Table 3).

**Table 3:** Multivariable logistic regression analysis for critical care and comfort care

	**Variables**	**Adjusted OR**^***c***^	**95% CI**	***p*-value**
**Critical care**^***a***^	Serious illness (No)	2.62	1.25–5.50	0.011
	Advance statement (No)	5.77	2.56–13.03	< 0.001
**Comfort care**^***b***^	Age (< 75 years)	2.62	1.42–4.83	0.002
	LST legal form documentation (Yes)	2.73	1.47–5.05	0.001

*Abbreviations*: *CI* Confidence interval, *KTAS* Korean Triage and Acuity Scale, *LST* Life-sustaining treatment, *OR* Odds ratio

^*a*^ Critical care was defined as receiving any of the following: cardiopulmonary resuscitation (CPR), mechanical ventilation (MV), renal replacement therapy (RRT), or extracorporeal membrane oxygenation (ECMO)

^*b*^ Comfort care was defined as receiving opioids for symptom relief within the last 24 h of life

^*c*^ The multivariable logistic regression model with stepwise, forward selection for both critical care and comfort care included age (< 75 or ≥ 75 years), sex, health insurance, status of serious illness (yes or no), status of cancer (yes or no), prior place before the visit, KTAS level (1–2 or 3–5), advance statement status (yes or no), status of legal form documentation regarding LST, and status of palliative center consultation

### Factors associated with comfort care

Using opioid administration in the last 24 h as an index, patients aged < 75 years (59.3% *vs.* 78.9%, *p* = 0.002; OR 2.56, 95% CI 1.41–4.64) and/or with advanced cancer (61.0% *vs.* 81.5%, *p* = 0.002; OR 2.81, 95% CI 1.46–5.42) received more comfort care. Patients with ACP conversations before/after ED visits received more comfort care than those without (29.8% *vs.* 36.7% *vs.* 4.0%, respectively, *p* = 0.005). However, comfort care was not affected by sex, type of health insurance, or place before ED visitation (Supplementary Table 3).

According to the multivariable analysis, patients aged < 75 years (adjusted OR 2.62, 95% CI 1.42–4.83) and/or with LST legal forms (adjusted OR 2.73, 95% CI 1.47–5.05) were more likely to receive opioids on their final day (Table 3).

### Comparing patients with/without cancer

Compared to patients without cancer, lower percentages of cancer patients were > 75 years of age (35.5% *vs.* 66.7%, *p* < 0.001) and/or female (29.8% *vs.* 70.2%, *p* = 0.004); 50.6% (41/81) of those without cancer had serious illnesses. There were no significant differences in KTAS (85.8% for levels 1–2 *vs.* 90.1% for levels 3–5, *p* = 0.352).

Cancer patients were significantly more likely to have advance statements (34.0% *vs.* 14.8%, *p* = 0.002) and LST legal forms (61.7% vs. 40.7%, *p* = 0.003). Contrastingly, few patients without cancer received palliative care consultations (2.5% vs. 19.2%, *p* = 0.003).

Cancer patients were significantly less likely to receive CPR (22.7% *vs.* 35.8%, *p* = 0.035), MV (28.4% *vs.* 49.4%, *p* = 0.002), and vascular access (arterial line, central access) (56.0% *vs.* 80.3%, *p* < 0.001), while patients without cancer were significantly less likely to receive opioids (18.5% *vs.* 39.0%, *p* = 0.002) (Supplementary Table 4).

## Discussion

The LST Decision Act has spurred interest in LST implementation [39–42], but data on ED patients at EOL is still lacking. The investigated patients received substantial critical care and insufficient comfort care even with improved ACP and documentation.

The decrease in the total number of ED visits in the current study is consistent with the previous ones [21–24]. It may be due to general hesitancy or ED transport refusal among patients [43, 44] and limited resources under new policies [24]. Although there were no significant differences in disease-related ED mortality by year, the increasing number of total deaths, length of stay, and proportion of patients with KTAS levels 1–2 is in line with worsening mortality in Korea which is affected by the collateral damage of COVID-19 [25]. Our finding that 63.5% of ED visitors had cancer supports a Taiwanese study that reported cancer patients visited EDs more frequently near EOL, possibly due to high national health insurance coverage [45].

The critical care rate decreased in the ED at EOL following the Act, but nearly 40% of patients still received it. This supports a study at the same institution that 12% and 37.8% of patients received CPR and MV upon terminal admission to general wards, ICUs, or the ED [39]. It is also similar to European EDs, with rates of 12.3%, 22.6%, and 12.3% for CPR, MV, and vasopressors, respectively [16]. In this study, many life-saving treatments other than CPR and MV were implemented in the final 24 h. Why is the rate of critical care so high despite large proportions of patients with cancer and serious illness? Supposedly, for ED physicians who primarily concentrate on resuscitation and reversibility, determining care goals at the first sight may be complex [32]. The pandemic may also have played a confounding role by adding uncertainties in resourcing and the goal of care. Even so, it is difficult to conclusive answer from the current study, thus, we should keep our attention on this issue.

Our finding that most patients started ACP conversations in the ED suggests opportunities to improve the rate [3, 46]. This is comparable to Western countries, where between 19 and 53% of patients complete AD upon ED visitation [47–49]. Nevertheless, this also shows that many patients do not make advanced EOL plans. Those with ACP conversations before ED visitation received significantly less critical care. Indeed, patients with cancer or serious illnesses receive less critical care due to better palliative care accessibility [50]. By contrast, physicians prioritize resuscitation over comfort care in patients with more clinical uncertainty. While physicians must not avoid intensive treatments when reversibility appears possible, they should also balance medical procedures to ensure adequate comfort [51]. With repeated clinical assessments, promptly clarifying the goal of care may lead to a chance for better care.

The increasing self-determination rates each year imply that the Act had positively affected autonomy, accordant with other studies [39, 40, 52], and it indicates improvement from the past when surrogates made most of the decisions [53]. Nevertheless, the family-determination rates remained higher than self-determination rates. For the family-determination, family members should overcome decisional conflicts they intensively experience near patients’ death [54, 55], and those decisions are often too late, resulting in insufficient ACP and more aggressive care [39]. In the meantime, about one-third of patients died without legal form documentation, and they received significantly more CPR or MV. However, we found no relevant differences in other medical procedures except antibiotics and opioids. Interestingly, some (i.e., lines, inotropics, high-flow nasal cannula) were performed more in those with legal forms. As such, legal documentation alone does not achieve the Act’s intent of protecting dignity and comfort. Unfortunately, except for a few experimental trials [56], there are no guidelines on appropriate EOL discussions in EDs. This current situation highlights the need for relevant frameworks to assist patients and caregivers.

Only 31.5% of patients received comfort care via opioids, with elderly patients and those without LST legal form documentation receiving significantly less. This is lower than in European EDs, where 55.3% received analgesics [16, 17], thus neglecting the desire to remain free of pain and anxiety during EOL [57]. However, environmental constraints make it unreasonable to conduct palliative care consultations with all ED patients. Here, suggested augmentations include screening criteria [58], training to recognize triggers [59], EOL management protocols [28], palliative care training courses [60, 61], and short-stay observation units [62]. ED physicians and palliative care specialists can also work toward a mutual understanding of their respective priorities, expectations, and management flow pressures [58, 63–66].

Our study has several limitations. First, this is a study from a single institution, SNUH, which contains a well-systemized palliative care center, thus limiting generalizability. Second, SNUH is renowned for treating cancer, with more than 60% of the sample affected; this is not representative of most EDs. Third, the retrospective design limited our understanding of the exact situations and depths of conversations on ACP and LST documentation.

## Conclusion

To the best of our knowledge, this is the first research to analyze EOL care in the ED after the enactment of the LST Decision Act in Korea. The Korean ED mortality rate is double that of Western institutions and interest in ACP is growing, so it is time to plan for better EOL care in the ED. We found that patients dying in the ED received much critical care, but insufficient comfort care influenced by ACP status, serious illness, and age. Therefore, physicians should minimize redundant evaluations and promptly introduce ACP to ensure better EOL care. These findings would help improve EOL care in the ED and guide healthcare professionals in reaching goal-directed EOL care provisions.

## Supplementary Information

**Additional file 1: Supplementary Figure 1.** Flow of eligible patients. **Supplementary Figure 2.** Status of general medical cares (procedures, evaluations, and medications) and critical cares (CPR, MV) in the emergency department in the last 24 hours of life by status of legal form documentation on life-sustaining treatment. **Supplementary Table 1.** Status of medical care in the emergency department within the last 24 hours. **Supplementary Table 2.** Status of advance care planning of patients who died in the emergency department by year. **Supplementary Table 3.** Factors associated with receiving comfort care at end-of-life in the emergency department. **Supplementary Table 4. **Comparisons between cancer and non-cancer patients.

## Data Availability

All data generated or analyzed during this study are included in this published article and its supplementary information files.
