# Supplementary material for: Aggressiveness of care in the last days of life in the emergency department of a tertiary hospital in Korea
Source: BMC Palliat Care. 2022 Jun 7;21:105. doi: 10.1186/s12904-022-00988-3 (PMC9170493; doi:10.1186/s12904-022-00988-3)
Supplement: Supplementary file 1 — Additional file 1: Supplementary Figure 1. Flow of eligible patients. Supplementary Figure 2. Status of general medical cares (procedures, evaluations, and medications) and critical cares (CPR, MV) in the emergency department in the last 24 hours of life by status of legal form documentation on life-sustaining treatment. Supplementary Table 1. Status of medical care in the emergency department within the last 24 hours. Supplementary Table 2. Status of advance care planning of patients who died in the emergency department by year. Supplementary Table 3. Factors associated with receiving comfort care at end-of-life in the emergency department. Supplementary Table 4. Comparisons between cancer and non-cancer patients. [file 12904_2022_988_MOESM1_ESM.docx]

**SUPPLEMENTARY**

**Supplementary Figure 1.** Flow of eligible patients

Abbreviations: ED, emergency department; *N*, number.


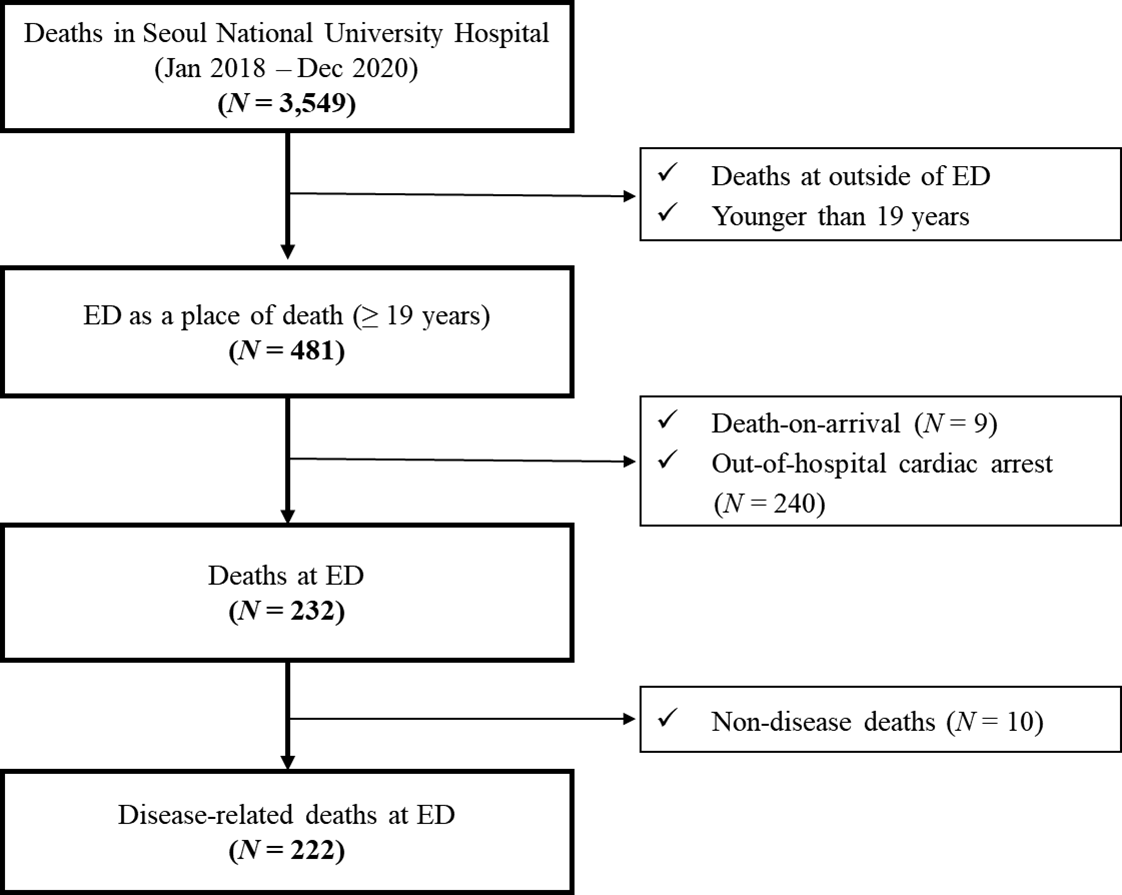


**Supplementary Figure 2.** Status of general medical cares (procedures, evaluations, and medications) and critical cares (CPR, MV) in the emergency department in the last 24 hours of life by status of legal form documentation on life-sustaining treatment.

Abbreviations: CPR, cardiopulmonary resuscitation; CT, computed tomography; CXR, chest radiograph; ECG, electrocardiogram; HFNC, high-flow nasal cannula; MRI, magnetic resonance imaging; MV, mechanical ventilation. **p*-value < 0.05


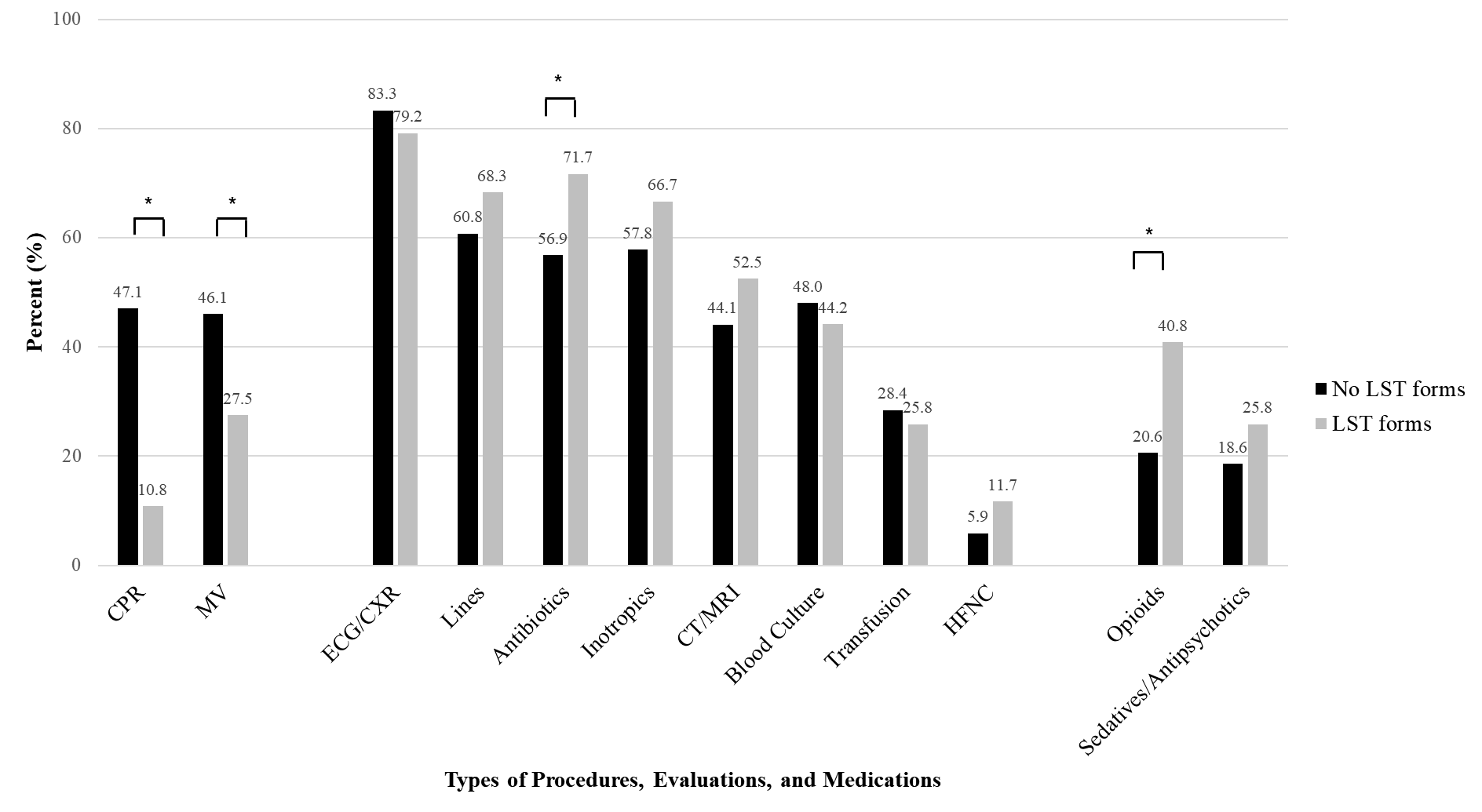


**Supplementary Table 1.** Status of medical care in the emergency department within the last 24 hours

|  | **Total (*N*=222)** | **2018 (*N*=41)** | **2019 (*N*=58)** | **2020 (*N*=123)** |  |
| --- | --- | --- | --- | --- | --- |
| Variables | **n (%)** | **n (%)** | **n (%)** | **n (%)** | ***p*-value^c^** |
| **Critical Care^a^** |  |  |  |  |  |
| No | 134 (60.36) | 23 (56.10) | 34 (58.62) | 77 (62.60) |  |
| Yes | 88 (39.64) | 18 (43.90) | 24 (41.38) | 46 (37.40) | 0.725 |
| **Types of Critical Care^b^** |  |  |  |  |  |
| CPR | 61 (27.48) | 16 (39.02) | 18 (31.03) | 27 (21.95) | 0.082 |
| MV | 80 (36.03) | 16 (39.02) | 21 (36.21) | 43 (34.96) | 0.895 |
| RRT | 1 (0.45) | 0 (0.00) | 0 (0.00) | 1 (0.81) | 1.000 |
| ECMO | 1 (0.45) | 1 (2.44) | 0 (0.00) | 0 (0.00) | 0.185 |
| **Procedure/Evaluation^b^** |  |  |  |  |  |
| ECG/CXR | 180 (81.08) | 34 (82.93) | 43 (74.14) | 103 (83.74) | 0.289 |
| CT/MRI | 108 (48.65) | 13 (31.71) | 31 (53.45) | 64 (52.03) | 0.055 |
| Arterial or central line | 144 (64.86) | 25 (60.98) | 23 (39.66) | 96 (78.05) | <0.001 |
| Routine lab | 205 (92.34) | 38 (92.68) | 52 (89.66) | 115 (93.50) | 0.660 |
| Blood culture | 102 (45.95) | 18 (43.90) | 27 (46.55) | 57 (46.34) | 0.958 |
| HFNC | 20 (9.01) | 2 (4.88) | 2 (3.45) | 16 (13.01) | 0.080 |
| **Medications^b^** |  |  |  |  |  |
| Transfusion | 60 (27.03) | 11 (26.83) | 14 (24.14) | 35 (28.46) | 0.830 |
| Antibiotics | 144 (64.86) | 19 (46.34) | 35 (60.34) | 90 (73.17) | 0.005 |
| Vasopressors | 139 (62.61) | 20 (48.78) | 34 (58.62) | 85 (69.11) | 0.051 |
| Opioids | 70 (31.53) | 9 (21.95) | 16 (27.59) | 45 (36.59) | 0.164 |
| Sedatives/Antipsychotics | 50 (22.52) | 7 (17.07) | 8 (13.79) | 35 (28.46) | 0.058 |

^a^ Critical care was defined as receiving more than any one of the followings: CPR, MV, RRT, or ECMO.

^b^ Critical care, procedure/evaluation, medications done within last 24 hours were counted, and multiple selections were allowed.

^c^ *p*-values were calculated using the Pearson's chi-squared test, but Fisher's exact test was used for RRT, ECMO, and HFNC.

Abbreviations: CPR, cardiopulmonary resuscitation; ECMO, extracorporeal membrane oxygenation; HFNC, high-flow nasal cannula; MV, mechanical ventilation; RRT, renal replacement therapy

**Supplementary Table 2.** Status of advance care planning of patients who died in the emergency department by year

|  | **Total  (*N*=222)** | **2018  (*N*=41)** | **2019  (*N*=58)** | **2020  (*N*=123)** |  |
| --- | --- | --- | --- | --- | --- |
| **Variables** | **n (%)** | **n (%)** | **n (%)** | **n (%)** | ***p*-value**^a^ |
| **Advance care planning conversation** |  |  |  |  |  |
| No | 25 (11.26) | 8 (19.51) | 9 (15.52) | 8 (6.50) | 0.055 |
| Before ED visit | 47 (21.17) | 5 (12.20) | 10 (17.24) | 32 (26.02) |  |
| After ED visit | 150 (67.57) | 28 (68.29) | 39 (67.24) | 83 (67.48) |  |
| **Advance statement** |  |  |  |  |  |
| No | 162 (72.97) | 37 (90.24) | 43 (74.14) | 82 (66.67) | 0.013 |
| Yes | 60 (27.03) | 4 (9.76) | 15 (25.86) | 41 (33.33) |  |
| Advance directives^†^ | 13 (21.67) | 0 (0.00) | 4 (26.67) | 9 (21.95) | 0.514 |
| POLST^‡^ | 47 (78.33) | 4 (100.00) | 11 (73.33) | 32 (78.05) |  |
| **Legal form documentation for LST implementation** | | | | | |
| None | 102 (45.95) | 37 (90.24) | 31 (53.45) | 34 (27.64) | < 0.001 |
| Patient self-determination | 50 (22.52) | 3 (7.32) | 11 (18.97) | 36 (29.27) |  |
| Family-determination | 70 (31.53) | 1 (2.44) | 16 (27.59) | 53 (43.09) |  |
| **Palliative care consultation** |  |  |  |  |  |
| No | 193 (86.94) | 37 (90.24) | 51 (87.93) | 105 (85.37) | 0.700 |
| Yes | 29 (13.06) | 4 (9.76) | 7 (12.07) | 18 (14.63) |  |

^a^ *p*-values were calculated using the Pearson's chi-squared test

Abbreviations: ED, emergency department; LST, life-sustaining treatment; POLST, Physician Orders for Life-Sustaining Treatment

**Supplementary Table 3.** Factors associated with receiving comfort care at end-of-life in the emergency department

|  | **No Comfort Care**  **(N=152)** | **Comfort Care^b^**  **(N=70)** |  | **Univariable  Logistic Regression** |
| --- | --- | --- | --- | --- |
| **Variables** | **n (%)** | **n (%)** | **p-value^c^** | **OR (95% CI)** |
| **Age (years), median (range)** |  |  |  |  |
| < 75 | 70 (59.32) | 48 (40.68) | 0.002 | 2.56 (1.41-4.64) |
| ≥ 75 | 82 (78.85) | 22 (21.15) |  | ref |
| **Sex** |  |  |  |  |
| Male | 94 (67.14) | 46 (32.86) | 0.579 | 1.18 (0.65-2.14) |
| Female | 58 (70.73) | 24 (29.27) |  | ref |
| **Health Insurance** |  |  |  |  |
| Medicaid/None | 15 (62.50) | 9 (12.86) | 0.505 | 1.35 (0.56-3.25) |
| National Health Insurance | 137 (69.19) | 61 (30.81) |  | ref |
| **Serious illness**^a^ |  |  |  |  |
| Yes | 120 (65.93) | 62 (34.07) | 0.083 | 2.07 (0.89-4.75) |
| No | 32 (80.00) | 8 (20.00) |  | ref |
| **Cancer (active)** |  |  |  |  |
| Yes | 86 (60.99) | 55 (39.01) | 0.002 | 2.81 (1.46-5.42) |
| No | 66 (81.48) | 15 (18.52) |  | ref |
| **Place prior to ED visit** |  |  |  |  |
| Home | 103 (67.32) | 50 (32.68) | 0.584 | 1.19 (0.64-2.21) |
| Others | 49 (71.01) | 20 (28.99) |  | ref |
| **KTAS level** |  |  |  |  |
| 3-5 | 16 (57.14) | 12 (42.86) | 0.168 | 1.76 (0.78-3.95) |
| 1-2 | 136 (70.10) | 58 (29.90) |  | ref |
| **Advance care planning conversation** | |  |  |  |
| After ED visit | 95 (63.33) | 55 (36.67) | 0.005 | 13.89 (1.83-105.56) |
| None | 24 (96.00) | 1 (4.00) |  | ref |
| Before ED visit | 33 (70.21) | 14 (29.79) |  | 10.18 (1.25-82.80) |
| **Advance statement** |  |  |  |  |
| Yes | 37 (61.67) | 23 (38.33) | 0.184 | 1.52 (0.82-2.83) |
| No | 115 (70.99) | 47 (29.01) |  | ref |
| **Legal form documentation for LST implementation** | | |  |  |
| Yes | 71 (59.17) | 49 (40.83) | 0.001 | 2.66 (1.46-4.86) |
| No | 81 (79.41) | 21 (20.59) |  | ref |
| **Palliative care consultation** |  |  |  |  |
| Yes | 15 (51.72) | 14 (48.28) | 0.037 | 2.28 (1.03-5.04) |
| No | 137 (70.98) | 56 (29.02) |  | ref |

^a^ Patients were considered to have serious illness if they were diagnosed as any of the followings (38): cancer with distant metastases, a chronic obstructive pulmonary disease with oxygen demand or in need of hospitalization, end-stage renal disease on dialysis, congestive heart failure in need of hospitalization, liver cirrhosis in Child-Pugh class C, diabetes with severe complications (ischemic heart disease, peripheral vascular disease, and renal disease), amyotrophic lateral sclerosis, or dementia with evidence of illness or advanced disease.

^b^ Comfort care was defined as receiving opioids for symptom relief within the last 24 hours of life.

^c^ *p*-values were calculated using the Pearson's chi-squared test for age, sex, health insurance, serious illness, cancer, prior place to ED visit, advance statement, legal form documentation and palliative consultation, or Fisher's exact test for ACP conversation.

Abbreviations: ECOG, Eastern Cooperative Oncology Group; ED, emergency department; EF, ejection fraction; FEV1, forced expiratory volume in one second; KTAS, Korean Triage and Acuity Scale; LST, life-sustaining treatment; NYHA, New York Heart Association

**Supplementary Table 4.** Comparisons between cancer and non-cancer patients

|  | **Total (*N*=222)** | **Non-cancer (*N*=81)** | **Cancer (*N*=141)** |  |
| --- | --- | --- | --- | --- |
| Variables | **n (%)** | **n (%)** | **n (%)** | ***p*-value^d^** |
| **Age (years), median (range)** |  |  |  |  |
| < 75 | 118 (53.15) | 27 (33.33) | 91 (64.54) |  |
| ≥ 75 | 104 (46.85) | 54 (66.67) | 50 (35.46) | <0.001 |
| **Sex** |  |  |  |  |
| Male | 140 (63.06) | 41 (50.62) | 99 (70.21) |  |
| Female | 82 (36.94) | 40 (49.38) | 42 (29.79) | 0.004 |
| **Health Insurance** |  |  |  |  |
| National Health Insurance | 198 (89.19) | 73 (90.12) | 125 (88.65) |  |
| Medicaid/None | 24 (10.81) | 8 (9.88) | 16 (11.35) | 0.734 |
| **Serious illness^a^** |  |  |  |  |
| No | 40 (18.02) | 40 (49.38) | 0 (0.00) |  |
| Yes | 182 (81.98) | 41 (50.62) | 141 (100.00) | <0.001 |
| **Place prior to ED visit** |  |  |  |  |
| Home | 153 (68.92) | 56 (69.14) | 97 (68.79) |  |
| Others | 69 (31.08) | 25 (30.86) | 44 (31.21) | 0.958 |
| **KTAS level** |  |  |  |  |
| 1-2 | 194 (87.39) | 73 (90.12) | 121 (85.82) |  |
| 3-5 | 28 (12.61) | 8 (9.88) | 20 (14.18) | 0.352 |
| **Advance care planning conversation** |  |  |  |  |
| None | 25 (11.26) | 15 (18.52) | 10 (7.09) |  |
| Before ED visit | 47 (21.17) | 7 (8.64) | 40 (28.37) |  |
| After ED visit | 150 (67.57) | 59 (72.84) | 91 (64.54) | <0.001 |
| **Advance statement** |  |  |  |  |
| None | 162 (72.97) | 69 (85.19) | 93 (65.96) |  |
| Yes | 60 (27.03) | 12 (14.81) | 48 (34.04) | 0.002 |
| **Legal form documentation for LST implementation** | | |  |  |
| None | 102 (45.95) | 48 (47.06) | 54 (52.94) |  |
| Yes | 120 (54.05 | 33 (27.50) | 87 (72.50) | 0.003 |
| **Legal form documentation for LST implementation** | | |  |  |
| None | 102 (45.95) | 48 (59.26) | 54 (38.30) |  |
| Patient self-determination | 50 (22.52) | 9 (11.11) | 41 (29.08) |  |
| Family-determination | 70 (31.53) | 24 (29.63) | 46 (32.62) | 0.002 |
| **Palliative care consultation** |  |  |  |  |
| None | 193 (86.94) | 79 (97.53) | 114 (80.85) |  |
| Yes | 29 (13.06) | 2 (2.47) | 27 (19.15) | **0.034** |
| **Types of Critical Care^b,c^** |  |  |  |  |
| CRP | 61 (27.48) | 29 (35.80) | 32 (22.70) | 0.035 |
| MV | 80 (36.03) | 40 (49.38) | 40 (28.37) | 0.002 |
| RRT | 1 (0.45) | 1 (1.23) | 0 (0.00) | **0.365** |
| ECMO | 1 (0.45) | 1 (1.23) | 0 (0.00) | **0.365** |
| **Procedure/Evaluation^c^** |  |  |  |  |
| CXR/ECG | 180 (81.08) | 68 (83.95) | 112 (79.43) | 0.408 |
| CT/MRI | 108 (48.65) | 38 (46.91) | 70 (49.65) | 0.695 |
| Arterial or central line | 144 (64.86) | 65 (80.25) | 79 (56.03) | <0.001 |
| Routine lab | 205 (92.34) | 75 (92.59) | 130 (92.20) | 0.915 |
| Blood culture | 102 (45.95) | 39 (48.15) | 63 (44.68) | 0.618 |
| HFNC | 20 (9.01) | 7 (8.64) | 13 (9.22) | 0.880 |
| **Medications^c^** |  |  |  |  |
| Transfusion | 60 (27.03) | 18 (22.22) | 42 (29.79) | 0.222 |
| Antibiotics | 144 (64.86) | 56 (69.14) | 88 (62.41) | 0.312 |
| Vasopressors | 139 (62.61) | 53 (65.43) | 86 (60.99) | 0.510 |
| Opioids | 70 (31.53) | 15 (18.52) | 55 (39.01) | 0.002 |
| Sedatives/Antipsychotics | 50 (22.52) | 22 (27.16) | 28 (19.86) | 0.210 |

^a^ Patients were considered to have serious illness if they were diagnosed as any of the followings (38): cancer with distant metastases, a chronic obstructive pulmonary disease with oxygen demand or in need of hospitalization, end-stage renal disease on dialysis, congestive heart failure in need of hospitalization, liver cirrhosis in Child-Pugh class C, diabetes with severe complications (ischemic heart disease, peripheral vascular disease, and renal disease), amyotrophic lateral sclerosis, or dementia with evidence of illness or advanced disease.

^b^ Critical care was defined as receiving more than any one of the followings: CPR, MV, RRT, ECMO during ED stay.

^c^ Critical care, procedure, evaluation, and medications performed within last 24 hours were counted, and multiple selections were allowed.

^d^ *p*-values were calculated using the Pearson's chi-squared test, but Fisher's exact test was used for palliative consultation, RRT, and ECMO.

Abbreviations: CXR, chest radiograph; CT, computed tomography; ECG, electrocardiogram; ECMO, extracorporeal membrane oxygenation; ED, emergency department; HFNC, high-flow nasal cannula; KTAS, Korean Triage and Acuity Scale; LST, life-sustaining treatment; MRI, magnetic resonance imaging; MV, mechanical ventilation; *N*, number; NYHA, New York Heart Association; RRT, renal replacement therapy
